# Virtual Experiments of Particle Mixing Process with the SPH-DEM Model

**DOI:** 10.3390/ma14092199

**Published:** 2021-04-25

**Authors:** Siyu Zhu, Chunlin Wu, Huiming Yin

**Affiliations:** Department of Civil Engineering and Engineering Mechanics, Columbia University, 610 S.W. Mudd, 500 West 120th Street, New York, NY 10027, USA; sz2507@columbia.edu (S.Z.); cw3056@columbia.edu (C.W.)

**Keywords:** smoothed particle hydrodynamics, discrete element method, Darcy’s law, homogeneity metric, particle mixing process

## Abstract

Particle mixing process is critical for the design and quality control of concrete and composite production. This paper develops an algorithm to simulate the high-shear mixing process of a granular flow containing a high proportion of solid particles mixed in a liquid. DEM is employed to simulate solid particle interactions; whereas SPH is implemented to simulate the liquid particles. The two-way coupling force between SPH and DEM particles is used to evaluate the solid-liquid interaction of a multi-phase flow. Using Darcy’s Law, this paper evaluates the coupling force as a function of local mixture porosity. After the model is verified by two benchmark case studies, i.e., a solid particle moving in a liquid and fluid flowing through a porous medium, this method is applied to a high shear mixing problem of two types of solid particles mixed in a viscous liquid by a four-bladed mixer. A homogeneity metric is introduced to characterize the mixing quality of the particulate mixture. The virtual experiments with the present algorithm show that adding more liquid or increasing liquid viscosity slows down the mixing process for a high solid load mix. Although the solid particles can be mixed well eventually, the liquid distribution is not homogeneous, especially when the viscosity of liquid is low. The present SPH-DEM model is versatile and suitable for virtual experiments of particle mixing process with different blades, solid particle densities and sizes, and liquid binders, and thus can expedite the design and development of concrete materials and particulate composites.

## 1. Introduction

Mixing of granular materials is crucial in a broad range of industrial processes, including constructional material production, advanced composite manufacture, mineral processing, plastics manufacturing, ceramic component, pharmaceutical tablets, and food products. Mix uniformity plays a critical role for the overall performance. Many experimental approaches have been employed in quality control of the mixing process, such as sampling, visual tracking, magnetic resonance imaging, rheometer measurements, etc. Among others, Khan et al. [1] used high speed image analysis to track the tracer particles, through which the dispersion coefficients are obtained according to fluctuating velocity components and partial mean-free path. Although these methods provide valuable insight of the mixing processes, their applications are limited due to the low consistency and high labor costs [2,3,4].

Moreover, it is time-consuming to develop a new particulate composite through such a trial-error method. Therefore, numerical simulation becomes an alternative to investigate granular flow dynamics.For solid applications, Orefice and Khinast applied DEM to study the transporting process of screw conveyors with different inclinations and filling levels [5]. And Sinaie simulated the size effects of concrete samples with DEM [6]. He et al. [7] investigated the interesting phenomenon of axial segregation and its key factors of the mixing process in a rotating drums. In [8], the authors extended the original DEM to an explicit model for identical superellipses that 2D discrete Fourier is applied to approximate the overlapping part explicitly. Among mixing problems, liquid-solid mixing becomes more common, and a reliable model to simulate the solid-liquid mixing process is highly demanded. Different approaches have been proposed to tackle the liquid-solid granular flow problems, which could be classified mainly to three categories: continuous media models, discrete particles models, and their combination [2,4,9].

Continuous media models treat both solid and liquid phases as interpenetrating continua. This scheme works well for problems with small deformation, but often fails in problems with large deformation, such as mixing problems. In comparison, discrete particle methods can simulate the dynamics of particulate mixtures and are capable of dealing with moving boundary problems in the mixing process, such as the Molecular Dynamic (MD), Discrete Element Method (DEM), Smooth Particle Hydrodynamics (SPH), and Dissipative Particle Dynamics (DPD). In these methods, some specific treatments are generally required to evaluate the coupling between solid particles and liquid particles [10,11,12].

Other methods combine continuous media and discrete particles, such as CFD coupling DEM [13,14], where CFD is implemented to simulate fluid as a continuum medium and DEM is applied to simulate solid particles as meshless discrete particles. For mixing problems where the solid load is high, but not high enough to apply liquid bridge models [15,16,17], the coupled CFD-DEM approach can be time consuming in modeling the moving boundary and discretizing the complex geometry, and SPH provides a practical method to simulate the liquid phase among a large number of solid particles with only a small number of liquid particles.

To tackle mixing problems with an intermediate to high solid load, SPH-DEM coupling is a promising approach. Cleary [12] proposed a one-way coupling SPH-DEM model to simulate slurry transport. Sun [18] introduced pressure difference and drag forces between liquid-solid interactions, and validated them with rotating drums filled with solid-liquid mixtures. Jonsen [19] employed the DEM stiffness to couple SPH with DEM, and obtained expected torque in a rotation drum; while Canelas [20] applied the SPH momentum equation to the interactions between solid and liquid particles and demonstrated the feasibility by comparing the simulation with the experiments in free surface solid-fluid flows. Robinson [21] introduced the local average N-S equation into coupling methods and validated the scheme with solid sedimentation in liquid. Robin’s scheme was later improved and applied to a high-shear mixing problem by Kwon [22] and Qi [23].

In comparison to DEM particles added to SPH liquid, some preliminary work has been conducted towards SPH liquid flow in porous media. Shao [24] and Zhu [25] treated porous structures as boundaries for SPH particles, which are in a smaller size compared to the porous structures. Jiang [26] proposed L-J potential between SPH and solid skeleton particles and demonstrated that the scheme can reproduce Kozeny’s formula of permeability. Peng [27] presented resistance interactions between solid and liquid SPH particles based on Darcy’s law and validated the method with a porous media case study.

However, it is still challenging for the coupled SPH-DEM method to simulate particle mixing with a high solid load due to the difficulty of calculating the interaction force between SPH and DEM particles, because most previous models were built under the assumption that solid particles are fully saturated by the liquid phase. This work aims at the solid-liquid mixing problems with very high solid loads, say up to 90% in volume. Here the key issue is how to calculate the interaction between liquid and solid particles. To compute the local liquid density, solid particles are considered to be rigid without any overlap with each other. Rather than excluding solid particles by dividing porosity in the density computation [21], this paper includes solid particles in density computation to avoid potential instability at a high solid load. SPH momentum equation is employed for the interaction between solid and liquid particles, in which the effective viscosity coefficient varies according to its local porosity following Darcy’s Law without using any artificial drag forces. This approach considers the nature of particle interactions in mesoscale, and is more efficient and effective for computation.

In the following, the numerical algorithm and formulation of the two-way DEM-SPH coupling method are introduced first. Two benchmark studies, i.e., (1) a solid particle sedimentation among liquid and (2) liquid flow through a porous media, are presented to verify the model for the parameter calibration. Subsequently, the present method is applied to high shear particle mixing problems, and a series of parametric studies are conducted. The local average mixing index is the main criterion to evaluate the homogeneity of the mixture, which will be used to optimize the particle mixing process for homogeneous mixtures.

## 2. Algorithm and Formulation

### 2.1. Smoothed Particle Hydrodynamics (SPH)

The Smoothed Particle Hydrodynamics (SPH) has been widely used for simulating fluid flows, since it was first developed by Gingold [28] initially for astrophysical problems. It was later used for simulating free surface flow [29] among other applications [30]. SPH is a mesh-free Lagrangian method, using particles to discretize continuous fluid field. “Smoothed” means that the property of the fluid field could be smoothed from discrete particles using a kernel function. Even though it is a particle based method, continuous fields of the fluid could be obtained by the weight average of neighbor particles through a kernel function. SPH could be applied to computer visualization and animation [31,32], as well as calculations on physical systems such as free surface flow [29], shock [33] and violent impact flows [34].

In an SPH model, each particle has its properties such as position, velocity, mass, pressure and energy. Pressure and temperature are functions of density and kinetic energy, respectively. During the flow process, the mass of each particle stays constant while the density keeps changing as the flow is assumed to be weakly compressible. For any variable f(x) at point *x*, SPH discretizes the domain to particles as follows:

(1) $$\begin{array}{c} f\left(x\right)=\int_{V}f\left(x^{\prime }\right)W\left(x-x^{\prime },h\right)dx^{\prime }=\int_{V}f\left(x^{\prime }\right)W\left(r,h\right)dx^{\prime } \end{array}$$

where $W(x-x^{\prime },h)$ is the kernel function with features of positive and monotonically decrease with distance [35] and $r=|x-x^{\prime }|$; *h* is the Kernel length which determines the interaction domain of the kernel function; *V* is the material domain. In this work, Cubic Spline kernel function [36] is implemented:

(2) $$\begin{array}{c} W\left(r,h\right)=A\left\{\begin{array}{cc} 1-1.5\times \frac{r^{2}}{h^{2}}+0.75\times \frac{r^{3}}{h^{3}}\; & \mathrm{if}\;r<h\\ 0.25\times {\left(2-\frac{r}{h}\right)}^{3}\; & \mathrm{if}\;h\leq r<2h\\ 0\; & \mathrm{if}\;2h\leq r \end{array}\right. \end{array}$$

where $A=\frac{1}{\pi h^{3}}$ for 3D simulation. The larger the value of *h*, the more the neighboring particles to be counted [37]. Different values of *h* are chosen for specific problems of interest considering a balance between accuracy and efficiency.The value of $W(x-x^{\prime },h)$ is zero when the distance between *x* and $x^{\prime }$ is larger than 2h, thus the effective integration domain is the material within a sphere with radius 2h centered at *x*. The density of each particle in SPH is calculated by the mass and kernel functions as follows:

(3) $$\begin{array}{c} \rho_{i}=\Sigma m_{j}W_{ij} \end{array}$$

where $W_{ij}$ = $W(x_{i}-x_{j})$. The governing equation of SPH are derived from the Navier-Stokes(N-S) equation as follows:

(4) $$\begin{array}{c} \frac{dv}{dt}=-\frac{1}{\rho }\nabla P+g+\nu \nabla^{2}v \end{array}$$

In practice, the continuity equation is automatically satisfied due to the principle of mass conservation of each particle [38]. Besides the N-S equation, SPH assumes that pressure is a function of density through equation of state. Among various equations of state, the Tait equation of state is widely used in fluid mechanics [29,35], which is expressed as:

(5) $$\begin{array}{c} P\left(\rho \right)=\frac{c_{0}^{2}\rho_{0}}{7}\left[{\left(\frac{\rho }{\rho_{0}}\right)}^{7}-1\right] \end{array}$$

The Tait equation has a parameter of sound speed $c_{0}$. This is the sound speed in the SPH fluid. It is usually chosen lower than the physical value, depending on the specific problem size to make the system more stable and yet maintain a low Mach number (lower than 0.1) in an incompressible flow [34,39]. To discretize the momentum equation, the commonly used momentum equation for particle *i* and particle *j* is applied

(6) $$\begin{array}{c} m_{i}\frac{dv_{i}}{dt}=-\Sigma m_{i}m_{j}\left(\frac{P_{i}}{\rho_{i}^{2}}+\frac{P_{j}}{\rho_{j}^{2}}\right)\nabla_{i}W_{ij}+F_{\mu }+F_{b} \end{array}$$

where

(7) $$\begin{array}{c} F_{\mu }=\Sigma m_{i}m_{j}\left(\frac{4\nu_{0}r_{ij}\cdot \nabla_{i}W_{ij}}{\left(\rho_{i}+\rho_{j}\right)\left(r_{ij}^{2}+0.01h^{2}\right)}\right) \end{array}$$

is laminar viscosity [40], $\nu_{0}$ is the kinematic viscosity of the liquid, and $F_{b}$ is body force that directly added to the particle on liquid, such as gravity. Because the liquid’s viscosity is relatively high in this work, laminar viscosity is used to represent the actual viscosity.

Notice that due to the approximation using discrete particles to represent a continuous flow, the modeling parameters in a particle method will not only depend on the fundamental material properties, but also change with particle size, modeling scale, and neighborhood cutoff-distance, among other factors. To obtain repeatable and reliable results, a procedure for the calibration and verification of the modeling parameters should be conducted before using the method in virtual experiments. Section 3 will demonstrate the procedure to select appropriate parameters based on the existing theoretical results.

### 2.2. Discrete Element Method (DEM)

Discrete Element Method (DEM) is a numerical method to simulate the motion and interaction of granular materials as discrete particles. The major physical law is the momentum conservation, i.e., Newton’s second law. The governing equations of DEM could be written as:

(8) $$\begin{array}{c} \begin{array}{cc} \frac{dr^{I}}{dt} & =V^{I},\;m^{I}\frac{dV^{I}}{dt}=F^{I},\;I^{I}\frac{d\omega^{I}}{dt}=T_{r}^{I} \end{array} \end{array}$$

where $I^{I}$, $\omega^{I}$, and $T_{r}^{I}$ stand for momentum of inertia, rotation speed, and torque of particle with index *I*, respectively. DEM particles have radius, and thus can have torque. He and Bayly coupled DEM and SPH to simulate interactions of liquid with solid phase [41]. In the methodology part, they considered the torque only for solid phase. Luding listed equation of DEM particles with absence of torque, and both of them obtained reasonable results [21]. Following the second one, the torque of DEM particles is not considered. Generally, DEM models could be classified to two types: hard-sphere and soft-sphere models. In the hard-sphere model, movements of particles are determined by momentum conservation. Only one collision is permitted at one time and it happens instantaneous; forces between particles are normally not explicit, and the hard-sphere model is mainly used in rapid granular flows [42]. The soft-sphere model allows small overlap (or deformation) of particles to calculate the elastic, plastic and frictional forces between them. The computational framework follows the similar way to the molecular dynamics (MD) simulation.

Among the existing soft-sphere models, there are various approaches proposed to solve the relation between particle overlap and interaction forces. The most common method is the linear spring-dashpot (LSD) model, in which the interaction between two particles is expressed by a normal spring and dashpot, a tangential spring and dashpot, and a torque [43]. It could be further simplified by only considering the spring force based on the relative velocity between particles to simulate the dashpot force [44]. The spring force part can resist against contraction and expansion of particle interactions, and the dashpot force part introduces friction and damps out the numerical scheme divergence. In DEM model, the underlying assumption is that all the particles are rigid sphere, and no deformation or bending is considered. Since the size of all particles are small and their shape is sphere, this assumption is reasonable. The damping force is used to stablize the numerical scheme and is the standard approach in DEM method. Other contact models for DEM simulations have also been proposed, such as Hertz [45], Mindlin and Deresiewicz theories [46]. Although the LSD model is the most widely used one in DEM simulations because of its simplicity and computational efficiency compared to those non-linear models, the selection of force parameters in LSD DEM formulation is crucial to the success of simulation results.

In the LSD modeling, there are two types of forces: normal ($F_{n}^{IJ}$) and tangential forces ($F_{t}^{IJ}$), which can be decomposed to a spring and a dashpot for elastic and dissipative forces, respectively, as follows

(9) $$\begin{array}{cc} F_{n}^{IJ} & =k_{n}\Delta r_{n}^{IJ}n^{IJ}-C_{n}V_{n}^{IJ} \end{array}$$

(10) $$\begin{array}{cc} F_{t}^{IJ} & =\left\{\begin{array}{cc} -k_{t}\Delta r_{t}^{IJ}t^{IJ}-C_{n}V_{t}^{IJ}\; & \mathrm{if}\;|F_{t}^{IJ}\left|\leq \mu \right|F_{n}^{IJ}|\\ -\mu |F_{n}^{IJ}|t^{IJ}\; & \mathrm{if}\;|F_{t}^{IJ}\left|>\mu \right|F_{n}^{IJ}| \end{array}\right. \end{array}$$

where $\Delta r_{n}^{IJ}=\left|\Delta r_{n}^{IJ}\right|$ and $\Delta r_{t}^{IJ}=\left|\Delta r_{t}^{IJ}\right|$ are normal and tangential displacements, respectively; $k_{n}$, $C_{n}$, and $k_{t}$, $C_{t}$ are spring stiffness and dashpot damping coefficients along normal and tangential directions, respectively, and μ is friction coefficient.

To reflect the physics correctly, it is important to use appropriate DEM parameters, and there are various approaches to predict these parameters analytically [47]. The normal damping coefficient $C_{n}$ can be determined analytically by normal spring stiffness $k_{n}$ and restitution coefficient *e*, as well as the friction coefficient μ, which are normally the inputs to the DEM simulation [48,49].

(11) $$\begin{array}{c} C_{n}=2\sqrt{m_{eff}k_{n}}\frac{\ln e_{n}}{\sqrt{\ln^{2}e_{n}+\pi^{2}}} \end{array}$$

where $m_{eff}=m^{I}m^{J}/\left(m^{I}+m^{J}\right)$ is the effective mass of particle *I* and *J*, and $e_{n}=1$ means pure elastic collision, while $e_{n}=0$ is for perfectly inelastic collision. Several existing works have been proposed to determine normal spring stiffness by matching the maximum strain energy [50], maximum normal overlap [47], and dimensionless contact duration [51] to the non-linear models. And μ and $e_{n}$ could be determined by directly applying their physical properties.

The tangential spring stiffness $k_{t}$ and damping coefficient $C_{t}$ can also be determined as follows [48]:

(12) $$\begin{array}{cc} k_{t} & =\frac{2}{7}k_{n},\;C_{t}=0.5C_{n} \end{array}$$

Although the parameters in the above force units have clear physical meaning, it is not straightforward to identify the appropriate value in the DEM simulation due to the significant simplification in material modeling, time integration, and geometric consideration. Therefore these parameters are also determined through comparing numerical results with classic contact mechanics models, such as the Hertz model, the Johnson-Kendall-Roberts (JKR) model, the Derjaguin-Muller-Toporov (DMT) model, etc., or calibrated by the experiments. For example, in many cases, the normal spring stiffness $k_{n}$, restitution coefficient *e*, and the friction coefficient μ are directly calibrated by the experimental results [47]. Therefore, a virtual experiment shall be set up with those parameters being calibrated and validated by the physical experiments.

### 2.3. Coupling between SPH and DEM

When liquid and solid particles coexist and interact with each other, the coupling force should be considered. In this work a two-way SPH-DEM coupling method is introduced. In contrast to one-way coupling where only one kind of particles exert force on the other, the two-way coupling means that both SPH particles and DEM particles exert forces on each other. Prior to computing the coupling force, the density of SPH particles needs to be revised to prevent overlap between DEM and SPH particles. To this end, one can introduce the concept of porosity, and then the volume fraction of liquid and solid phase are specified [21]. For an arbitrary particle *i*, its porosity Po is computed as below:

(13) $$\begin{array}{c} Po_{i}=\frac{\Sigma_{liquid}V}{\Sigma_{all}V} \end{array}$$

Then the density of the SPH liquid particle is calculated by the weighted sum of its neighbor particles divided by its porosity:

(14) $$\begin{array}{c} \rho_{i}=\frac{\Sigma m_{j}W_{ij}}{Po_{i}} \end{array}$$

In this way, when a DEM particle enters the neighborhood of a SPH particle, the porosity of this SPH particle decreases, and its density increased, generating repulsive forces between its original neighbor SPH or DEM particles (since pressure is an increasing function of density, as mentioned before). When these neighbor particles reach balance again, the number of SPH or DEM particles in the SPH particle’s region is decreased, thus making room for the entering DEM particle and no particles are overlapped during this process.

However, at mixing cases where the solid load is as high as 80% to 90%, the porosity of SPH particles is inevitably low, which makes the computation of liquid density sensitive to the changes of its position and easy to become unstable. To solve this problem, a new method to compute the SPH particle’s density is proposed as below:

(15) $$\begin{array}{c} \rho_{i}=\Sigma_{liquid}m_{j}W_{ij}+\Sigma_{solid}m_{j}W_{ij} \end{array}$$

where the mass of solid particles is included in the computation of SPH particle’s density because the solid particles play a dominant role for the mix at a high solid load. Note that the mass of solid particles in the above equation is not exactly the solid mass, but the mass of liquid in the same amount of the solid’s volume. In other words, solid particles are treated as liquid while computing SPH particles’ density. In this approach, no overlap between solid and liquid particles will appear, and the scheme is still stable for even high solid loads, because there is no large fluctuations of SPH particle’s densities during the simulation process.

As for the coupling forces between DEM and SPH particles, one common approach is to apply buoyancy or pressure gradient forces and drag forces on DEM particles, and then apply the counteractive forces on SPH particles to attain two-way coupling. This approach is based on the assumption that solid particles “float” in liquid, in which liquid take the majority of the space. However, when the solid load is high, the assumption that solid particles are fully saturated by liquid particles does not hold anymore. In this work, a more fundamental formulation is derived and the coupling forces are still comprised of two parts, i.e., pressure gradient force and viscous force. For the pressure gradient force, the formula is the same as the SPH pressure gradient force, i.e., treating part of the solid-liquid interaction as SPH pressure gradient forces. Pressure gradient forces make SPH and DEM particles stable and do not overlap, while the essential coupling mechanism is the viscous force.

In flows among porous media, when the solid portion of the porous media is high, its macroscopic behavior can be described by Darcy’s Law [52]:

(16) $$\begin{array}{c} v=\frac{k}{\mu }\nabla P \end{array}$$

where *v* is the velocity of the flow, *k* is the permeability of the porous media, μ is the viscosity of the flow, ∇P is the pressure gradient. Darcy’s Law describes a fluid flow through a porous medium, which was formulated by Henry Darcy based on the results of experiments [53]. SPH is a Lagrangian fluid dynamics method as it traces motion of certain coordinates of particles. In Equation (4), the Lagrangian time derivative become 0 due to its weakly compressible feature. The viscous force in SPH depends on the local density and distribution of neighbor particles, and it changes linearly with the velocity difference, which matches Darcy’s law. Since pressure gradient force is proportional to ∇P, at equilibrium state it could be seen that the viscous force $F_{d}$ follows:

(17) $$\begin{array}{c} F_{d}∼\frac{v\mu }{k} \end{array}$$

In this scheme, since permeability *k* is a function of porosity, the viscous force between DEM and SPH particles is a function of porosity. When the liquid load is high enough in the simulation, the interactive viscous force between DEM and SPH particles will be close to the viscous force between the SPH particles. Depending on the porosity of the target particles, the coupling viscous force will be multiplied by a coefficient, which is a function of porosity, and satisfies the classical Darcy’s Law [25,26,52]:

(18) $$\begin{array}{c} f_{ij}^{d}=\lambda f_{ij}^{d,SPH} \end{array}$$

where

(19) $$\begin{array}{c} \lambda =1+\frac{C\Phi^{2}}{{\left(1-\Phi \right)}^{3}r^{2}} \end{array}$$

in which Φ is the volume fraction of the solid. Φ is calculated for all the liquid particles, and for a liquid particle *i*, its Φ is calculated as

(20) $$\begin{array}{c} \Phi =\frac{\Sigma_{solid}m_{j}W_{ij}}{\Sigma_{liquid}m_{j}W_{ij}+\Sigma_{solid}m_{j}W_{ij}} \end{array}$$

and $f_{ij}^{d,SPH}$ is the drag force between particles *i* and *j* that calculated using Equation (7), the SPH viscous equation. When Φ is 0 it is pure liquid, λ=1. When Φ is high, λ>>1, and the value of λ is approximately proportional to $\frac{\Phi^{2}}{{\left(1-\Phi \right)}^{3}r^{2}}$, which is the classical result observed in experiment [27]. And *C* is a parameter to be determined by experiments too.

### 2.4. Boundary Conditions

For DEM particles, their interactions with boundary could be simulated using the LSD model, where the forces are computed through the overlap between particles and boundary. The relevant parameters could be determined analytically or through experiments.

For SPH particles, it is not straightforward to set boundary conditions in particle dynamic simulations, because when SPH particles approach a rigid boundary, the support domain of the SPH particles in the kernel function is cut off by the boundary [54]. Periodic boundary condition can avoid this problem. For fixed boundary conditions, one way is to set dummy particles to approximate the interface between the fluid phase and the boundary. The dummy particles will be counted to update normal particle’s density, but their position and velocity will not be updated. They are predefined to be fixed at the boundary. In this study, to be consistent, SPH particles share the same boundary conditions as DEM particles.

### 2.5. Stability

For DEM particles, the critical time step, or the collision duration is expressed as below:

(21) $$\begin{array}{c} t_{c,n}=\pi \left(\frac{k_{n}}{m_{eff}}-\frac{C_{n}^{2}}{4m_{eff}^{2}}\right) \end{array}$$

In convention, the time step is chosen as $\Delta t=min(t_{c,n}/50)$ to maintain the stability of the simulation. To approach the physical values, the time step Δt has to be very small, which makes the simulation very time consuming. On the other hand, sometimes the normal spring stiffness does not play a crucial role towards the simulation results so it could be reduced to increase the critical time steps. Considering these two aspects, usually $k_{n}$ is determined in the scenario when the maximum overlap between two solid particles is less than 1% of their diameter, and in this way the computation efficiency of DEM simulation is substantially improved [55,56].

The main requirement for the time step in SPH is that particles do not travel through its neighbor particles in one time step [38], which leads to a criterion as

(22) $$\begin{array}{c} t_{1}=\min\sqrt{\left(\frac{h}{f_{k}}\right)} \end{array}$$

where $f_{k}$ stands for the resultant force associated with particle *k*.

In addition, during each time step a wave does not travel out of the domain [57], which leads to another criterion as follows:

(23) $$\begin{array}{c} t_{2}=\min\frac{h}{c_{s}+h\max\frac{u_{k,l}x_{k,l}}{x_{k,l}^{2}}} \end{array}$$

where $c_{s}$ is the speed of sound in simulation, $x_{k,l}$ and $u_{k,l}$ are position and relative velocity between particles *k* and *l*.The condition for global stability constraint is the combination of both criteria:

(24) $$\begin{array}{c} \Delta t=C\min(t_{1},t_{2}) \end{array}$$

where *C* is the Courant number, chosen in the range of 0.1 to 0.3. For a coupling problem, the time step should satisfy both DEM and SPH requirements.

### 2.6. The Mixing Index

During the mixing process, the particle distribution keeps changing until a relatively uniform distribution is achieved. It is therefore necessary to quantify the mixing state. In this work the local average index is implemented to test the homogeneity of the mixture. In this method, a 3D grid will be constructed over the chosen domain. The size of each cell in the 3D grid, i.e., the Representative Volume Element(RVE) should be at least five times of the average particle diameter [55] so that the local averages of a selected properties, such as mass, density, diameter, etc., are statistically meaningful. Then the local average values will be calculated using all the particles within the cell, so that the probability distribution for the local averages can be constructed. Afterwards, the mean, standard deviation, and coefficient of variation η (defined as mean divided by standard deviation) will be computed over the entire domain of local cells at each time step. In this work, the number fraction of certain type of particles in each RVE is chosen as the target value to test, and its value in each RVE and its variance among all the RVEs are calculated. If the mixture is homogeneous, the number fraction of any type of particles will be similar in each RVE and the variance among all the RVEs is low. Two ideal limits will also be calculated for η for the fully segregated $\eta_{s}$ and fully mixed states $\eta_{r}$, so that η can be normalized to the range of 0–1 to characterize the quality of particles mixing which is expressed as

(25) $$\begin{array}{c} \xi =\left(\eta -\eta_{r}\right)/\left(\eta_{s}-\eta_{r}\right) \end{array}$$

where ξ=0 indicates a fully mixed states; while ξ=1 suggests a fully segregated state. The local average index is not only a good indicator of the homogeneity of the final mixture, but also a natural measure of the mixing rate. Moreover, this method is also very effective in the experiment perspective. Depending on the sizes of the particles in the mix design, different RVEs could be chosen to test the mixing quality.

### 2.7. Particle Dynamics Parallel Simulator (PDPS)

The simulation in this work is done by software package Particle Dynamics Parallel Simulator (PDPS), developed in the Pao Sustainable Engineering and Materials Laboratory (Pao Lab) of Columbia University. It was inspired and designed based on the structure from the open source molecular dynamics software LAMMPS (http://lammps.sandia.gov/index.html, version: 16 March 2018). When observing the lack of available sources to conduct particle dynamics simulation with various particle based potentials, such as SPH, DEM, dissipative particle dynamics (DPD) [58], etc., we were motivated to provide a general platform for researchers interested in particle dynamics simulation and virtual experiments of large particle systems.

**Figure 1:**
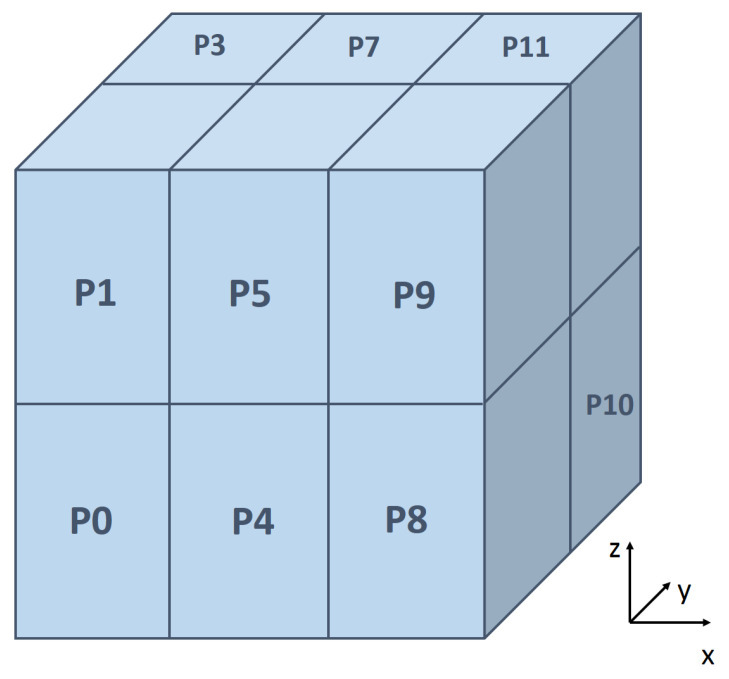
A schematic illustration of the domain decomposition technique.

PDPS is written in C++, and it uses Message Passing Interface (MPI) to conduct the parallel computing in a distributed memory environment. It can be run on both a single processor or multiple processors machine which can compile C++ and support the MPI library. The parallelism is fulfilled by using the domain decomposition technique, which has been demonstrated as one of the most efficient parallel algorithm towards molecular dynamics simulation [59]. A schematic illustration of the domain decomposition is shown in Figure 1, in which the entire domain is discretized into 3D grids and each grid is assigned to a processor. Only particles inside the local domain belongs to the corresponding processor, and every few time steps the particle list is updated as certain conditions are triggered. In high shear mixing problems, most of the particles are packed uniformly at the bottom of the mixer container because of gravity, which makes the decomposition of subdomains easy and MPI parallel scheme is efficient.

**Figure 2:**
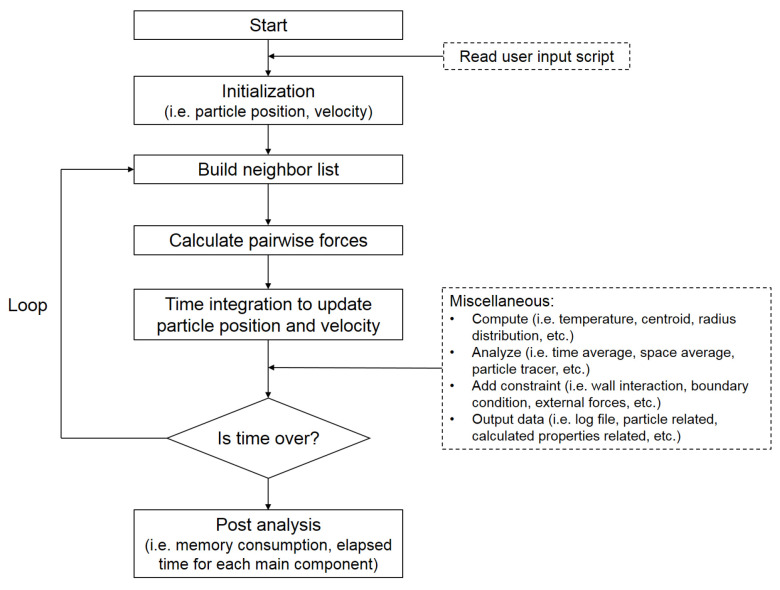
A flow chart of the computational steps with PDPS.

Similarly to LAMMPS, the architecture of PDPS includes the following modules: (1) initialize the computational model; (2) build the list of neighbor particles; (3) calculate pairwise forces between particles based on the neighbor list (4) time integration (i.e., velocity-Verlet algorithm) to update particle position and velocity; (5) repeat step (2) if simulation is not finished. Pairwise force computation is the most time consuming part, thus the time complexity of the particle dynamics simulation problem is O(n×k), where *n* is the total number of particles simulated, and *k* is the average number of neighbor particles that each particle has, typically around 100. A flow chart is provided in Figure 2.

## 3. Benchmark Studies

One major concern of particle dynamics methods is how to choose simulation parameters correctly. In this work, the parameters in simulation is chosen from classical benchmark studies. Parameters used in the SPH-DEM model are chosen to match some classical solid-liquid two phase flow problems, and these parameters will be applied to solid-liquid mixing problems.

### 3.1. A Single DEM Particle Falling in a Fluid

**Figure 3:**
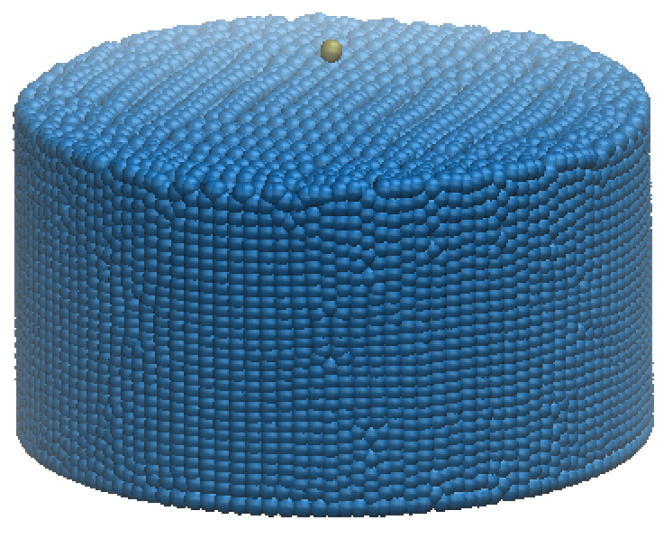
Sedimentation of a single DEM particle among SPH liquid particles.

In this case, a single DEM solid particle falling in a fluid of many SPH particles is investigated. This test is the first step to validate the feasibility of the DEM-SPH coupling method. As shown in Figure 3, the simulation domain is a cylinder tank with radius 20 cm, height 30 cm.

Properties of DEM and SPH particles are listed in Table 1. In Table 1, *h* is the smoothing length of SPH particles, and it is considered as the radius of SPH particles. The smoothing length is defined the same way in the following case studies. Since the Re number in this case is less than 1, it could be considered as a Stokes flow.

Based on the Stokes flow equation, the velocity of this single DEM particle could be expressed as:

(26) $$\begin{array}{c} V=\frac{2\Delta \rho gR^{2}}{9\mu }=0.218\;m/s \end{array}$$

**Figure 4:**
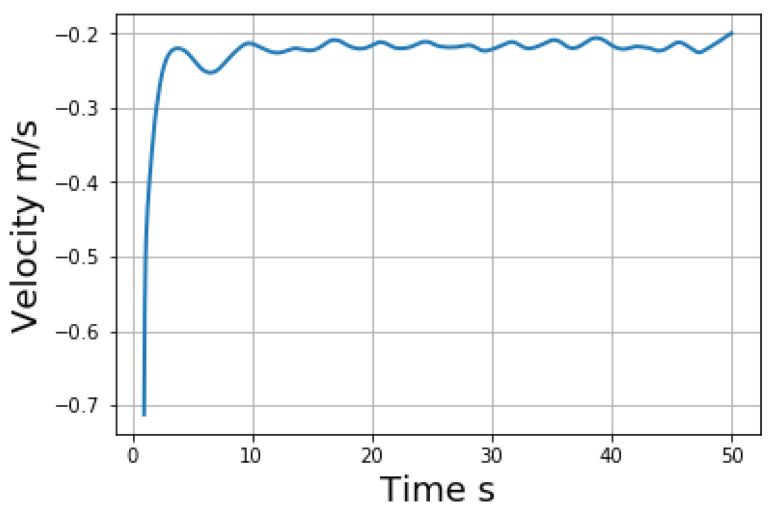
Sedimentation velocity of a single DEM particle among SPH particles.

Since the coupling forces between DEM and SPH particles have a range of interaction domain rather than stay on a distinct radius, a multiplier is applied to the viscosity forces between DEM and SPH particles to get the correct Stokes flow velocity. It is found when the multiplier is around 3.08, the Stokes flow could be reproduced as shown in Figure 4. Since the particle drops from above and hits its neighbor particles at the beginning, it is reasonable that the dropping velocity exhibits some oscillations. The oscillation will be smoothed out when all the neighbor particles get stable. Notice the fundamental material parameters at the continuum level need to be tuned at the coarse grain level [58]. Kumar et al. [60] provided a detailed explanation on the coarse-grained parameter mapping. This paper follows the common procedure to use established analytical solution to tune the parameters for partice mixing simulation.

In this example for sedimentation of a single DEM particle among SPH liquid particles, the coupling force can effectively capture the physics of the interaction between liquid and solid particles. Notice that the surface toughness and surface tension of the particle may play an important role for the particle sedimentation [61], this benchmark case considers the perfectly smooth surface without surface tension.

### 3.2. Liquid Flowing through a Porous Media

**Figure 5:**
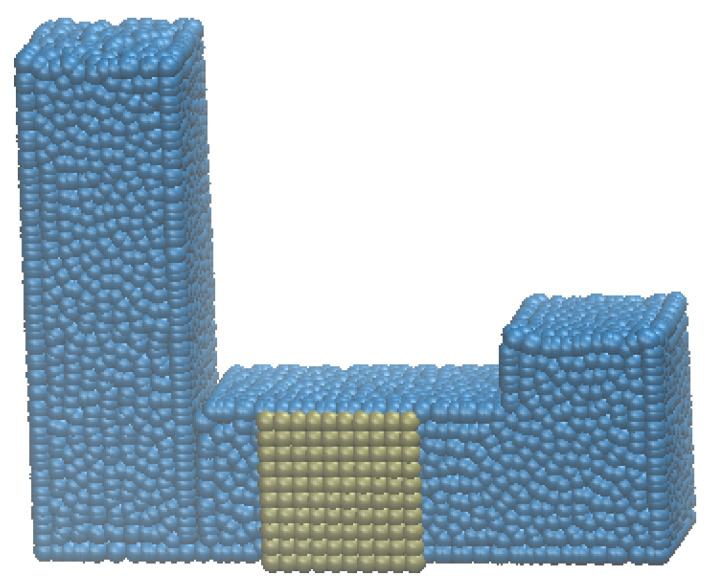
SPH liquid flow through porous media.

The second case study evaluates the effectiveness of the present method on a more complicated scenario, liquid flowing through a porous media. In this case a U-tube with a square width of 10 m and a horizontal length of 60 m is generated, as shown in Figure 5. The vertical height is different between the two sides. In the middle of the tube filled in a cubic porous media with DEM particles accounting for 52.3% of the total volume of the cubic media.

The properties of SPH and DEM particles are listed in Table 2.

**Figure 6:**
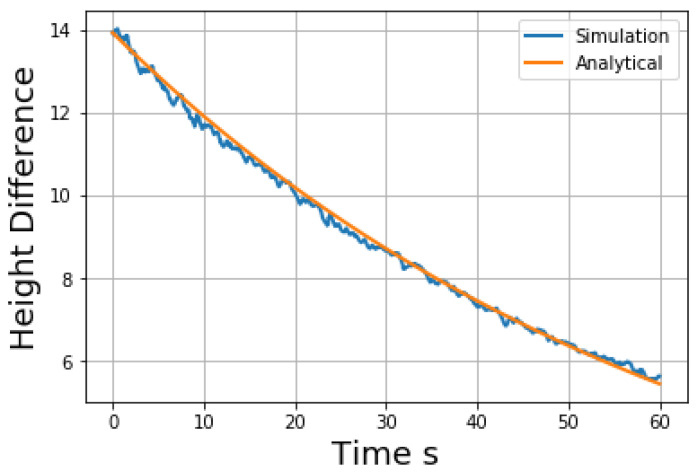
Height difference of a SPH liquid flow through a porous media.

As the liquid flowing from one side of the U-tube to the other side, the flow speed and the height difference between the two sides depend on the porosity of the porous media and viscosity of the liquid. Based on previous studies [27], the height difference between two sides will show an exponential trend:

(27) $$\begin{array}{c} \Delta H=\Delta H_{0}exp\left(-\frac{2K_{h}t}{L}\right) \end{array}$$

(28) $$\begin{array}{c} K_{h}=\frac{\rho gk}{\mu } \end{array}$$

(29) $$\begin{array}{c} k=\frac{Cr^{2}{\left(1-\phi \right)}^{3}}{\phi^{2}} \end{array}$$

where ϕ is the volume fraction of the solid phase, and *C* is a problem dependent parameter to be determined. As shown in Figure 6, the height difference between two sides of the U-tube follows an exponential curve in simulation, which shows that the present model catches this physics. Here $C=8.07\times 10^{-7}$ is determined by matching the simulation results with the analytical solution, and this value is used in the following simulation studies. The unstable value of particle velocity is caused by the movement of its neighbor particles, since the target particle is dropping from above. It becomes stable along with time.

## 4. Numerical Simulation and Results

### 4.1. Simulation Setup

**Figure 7:**
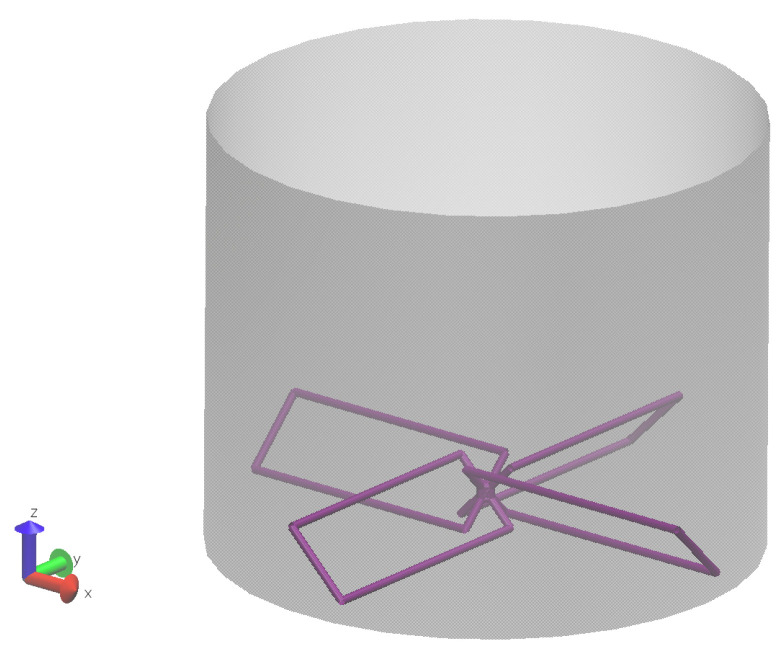
Snapshot of the high shear mixing simulation mixer.

After the successful demonstration of the present method in the two case studies, it is applied to the simulation of a solid-liquid mixing process in a high shear mixer with four blades. As shown in Figure 7, in a cylinder mix container, four rectangular plane blades are placed at the bottom with uniform angles. The geometry of this mixer is listed in Table 3. To visualize these four blades, particles are created along the boundaries of these blades. The interaction of particles with the four blades is the same as their interaction with the cylinder container, which is Linear Spring-Dashpot (LSD) method with given stiffness and dashpot parameters.

In the simulation conducted below, two types of solid particles and one type of liquid particles are employed to investigate the mixing performance of solid-liquid two-phase flow. The properties of the two types of solid particles are the same except color, which is used to demonstrate the mixing process. Their properties are listed in Table 4. As mentioned before, the sound speed here is chosen as 30 m/s to increase the time step, while maintaining a low Mach number (The velocity at the tip of the blades is around 2 m/s, and the maximum velocity of particles could not exceed that, so the Mach number is below 0.1). It is a common practice to choose the sound speed as 5–20 times the maximum velocity of particles in the simulation [62,63].

**Figure 8:**
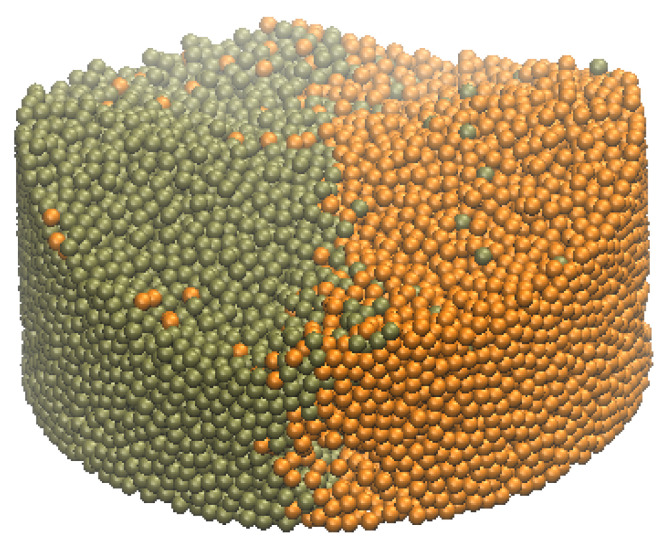
Initial state of mixing problem.

Prior to mixing, the solid particles are classified into two groups (the orange and tan colors, respectively), with each group filling half side of the container, as shown in Figure 8. The two types of particles are first created and then released to fill up the space in the container, so there are a few particles scattered into the other type of particles during the falling process.

Then liquid particles are generated at the top of these solid particles. After these particles reach stable, the blades start to rotate with a constant acceleration rate until a given velocity is reached. Then the two groups of solid particles together with liquid particles are mixed towards homogeneous distribution until the rotation of the blades stopped. The liquid acts like a binder to make two groups of solid particles bound together. It is similar to the cohesive effects in liquid bridge model, but here SPH liquid particles could deal with a high liquid portion (>10%). In the following parts, the mixing process is analyzed in detail, and the effects of different variables such as the amount of liquid content, viscosity of liquid on the mixing performance are investigated.

### 4.2. Mixing Process Analysis

**Figure 9:**
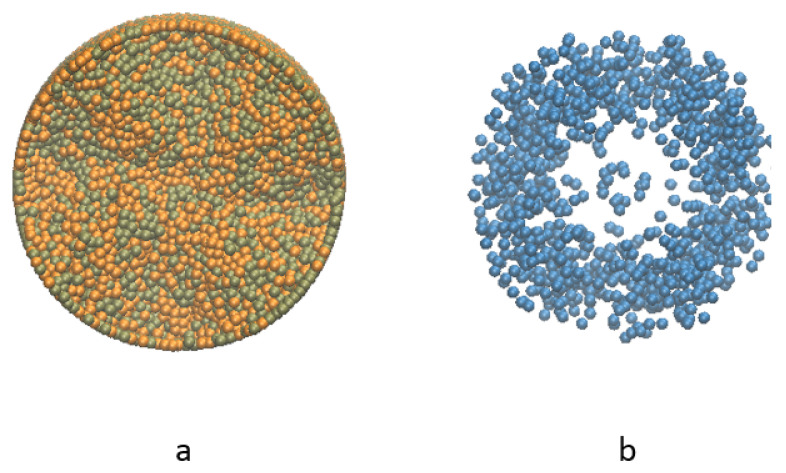
Topview of particle distribution after mixing (**a**) solid particles and (**b**) liquid particles.

Since at the beginning two kinds of solid particles are placed in symmetry, the radial distribution and height distribution of the two types of solid particles are expected to be comparable after certain time of mixing as shown in Figure 9a. However, there are still some small aggregates in the mixture, which is caused by the strong viscous force of the liquid particles inside, shown in Figure 9b.

**Figure 10:**
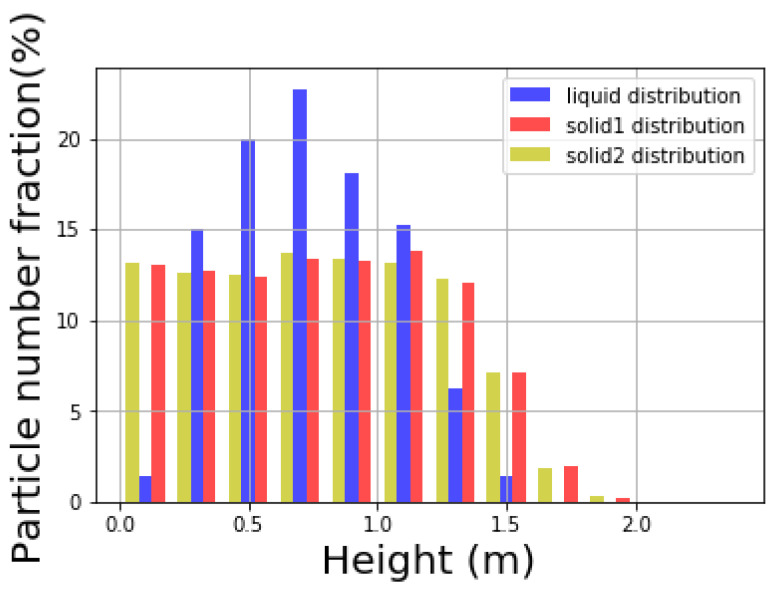
Liquid distribution on height.

**Figure 11:**
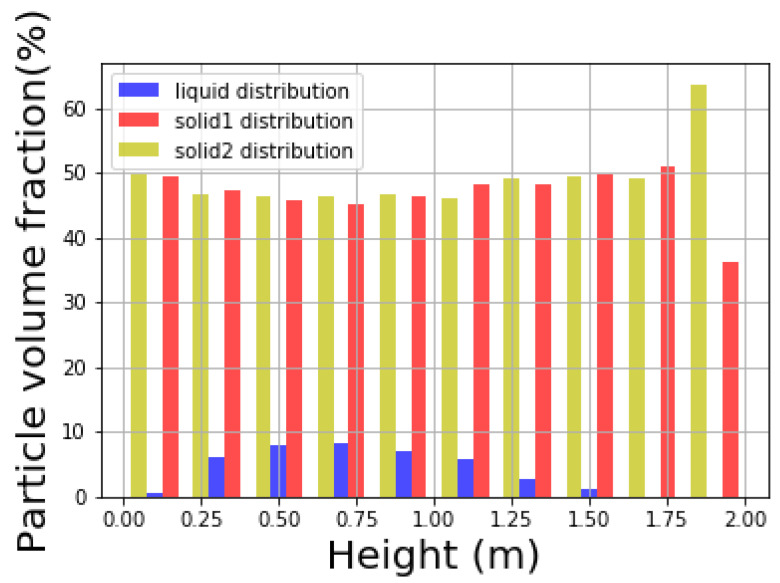
Particle volume fraction on height.

In addition to the top view, the distribution of liquid at different height of the cylindrical container is expressed in Figure 10 and Figure 11. Figure 10 shows the distribution of different types of particles at different height levels, and the sum of all the distributions of certain type equals to 1. Figure 11, on the other hand, shows the volume fraction of different types of particles at certain height level, and the sum of all the volume fractions at every height level equals to 1.

**Figure 12:**
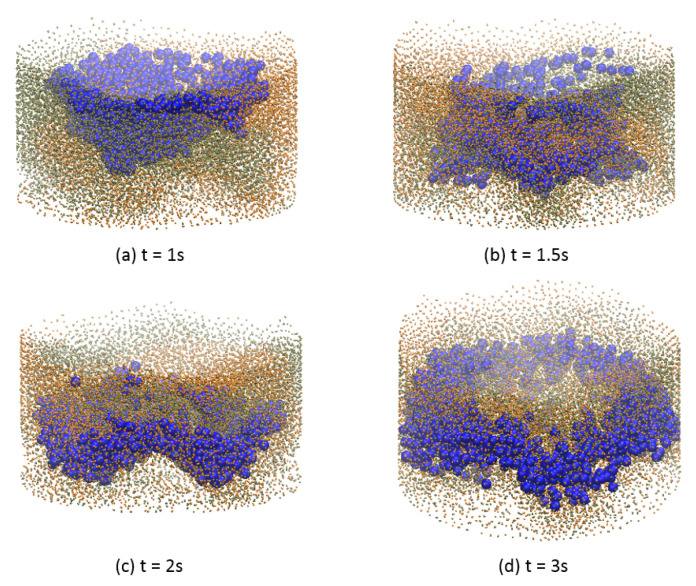
Liquid distribution evolution during mixing process.

Notice that while the solid particles are distributed homogeneously along the vertical direction of the cylinder, the liquid mainly stays in the middle range. Therefore, the liquid is unable to be mixed well vertically within the solid particles in the current configuration. In Figure 12 the distribution of liquid particles in the whole mixture during the mixing process is illustrated. Solid particles that are in orange and tan are plotted smaller purposely to let those blue liquid particles easy to identify. It is shown that liquid particles mainly lie in the middle of the mixture vertically at the end of the mixing process. Due to resolution issue, it looks like some part of the mixture is not covered by liquid particles. If enough liquid particles are used in the simulation, those parts will be covered by liquid particles.

### 4.3. The Effect of Liquid Content on Mixing Property

**Figure 13:**
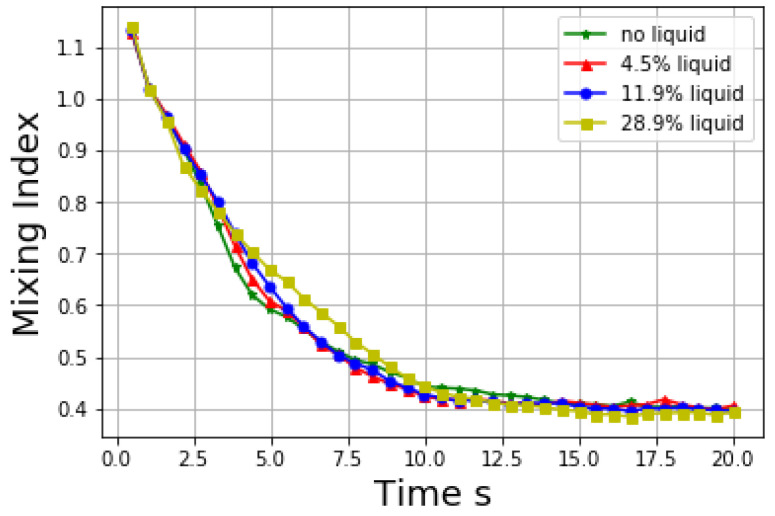
Mixing index at different liquid amount.

In this part, the effect of liquid amount on mixing performance is studied. To test the mixing quality, local average mixing index is adopted here to examine the homogeneity of the particles with a cubic RVE of size 30 mm × 30 mm × 40 mm. In the following case studies, the mixing index is defined the same way. In this part, the effect of liquid amount on mixing performance is studied and the corresponding results are shown in Figure 13.

**Figure 14:**
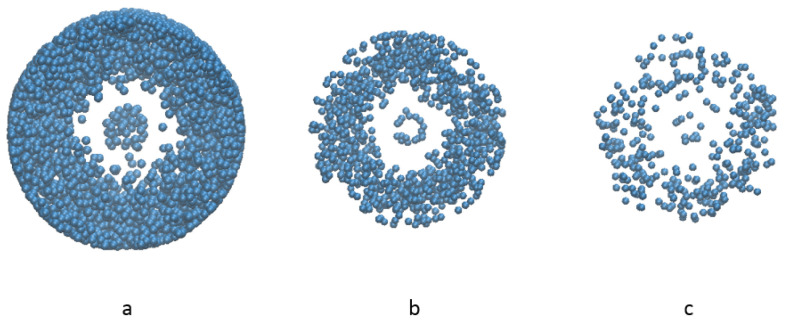
Liquid distribution comparison of different liquid contents (**a**) 28.9% liquid (**b**) 11.9% liquid (**c**) 4.5% liquid.

Top view of the distribution of liquid particles is shown in Figure 14. Generally, as the liquid content decreases, the distribution of liquid particles is similar, and the mixing of solid is not influenced much at the viscosity value 0.2 m${}^{2}$/s. However, when the liquid amount is as high as 28.9% the mixing process is slightly slower than other cases, and it thus takes a longer time for the mixing index to reach the asymptotic value).

### 4.4. The Effect of Liquid Viscosity on Mixing Property

**Figure 15:**
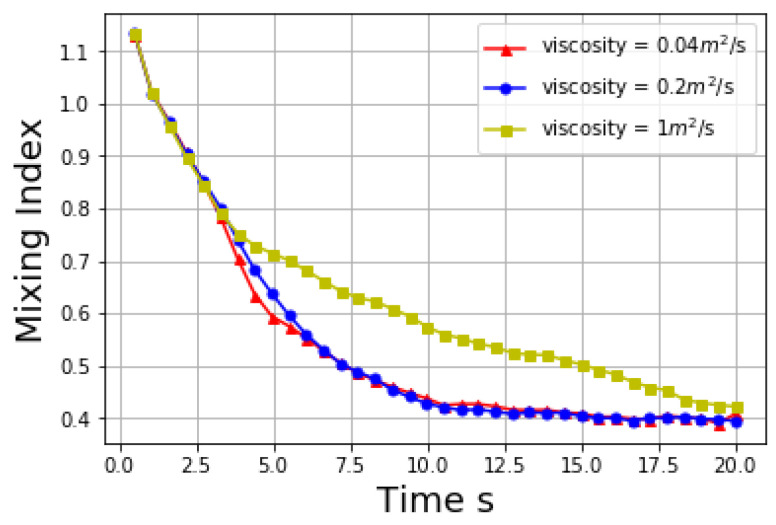
Mixing index at different viscosity.

The viscosity of liquid has a significant effect on the mixing performance. Three different kinematic viscosity 0.04 m${}^{2}$/s, 0.2 m${}^{2}$/s and 1 m${}^{2}$/s are applied while other parameters keep the same. The evolution of mixing indices with time in these simulations is listed in Figure 15 below.

**Figure 16:**
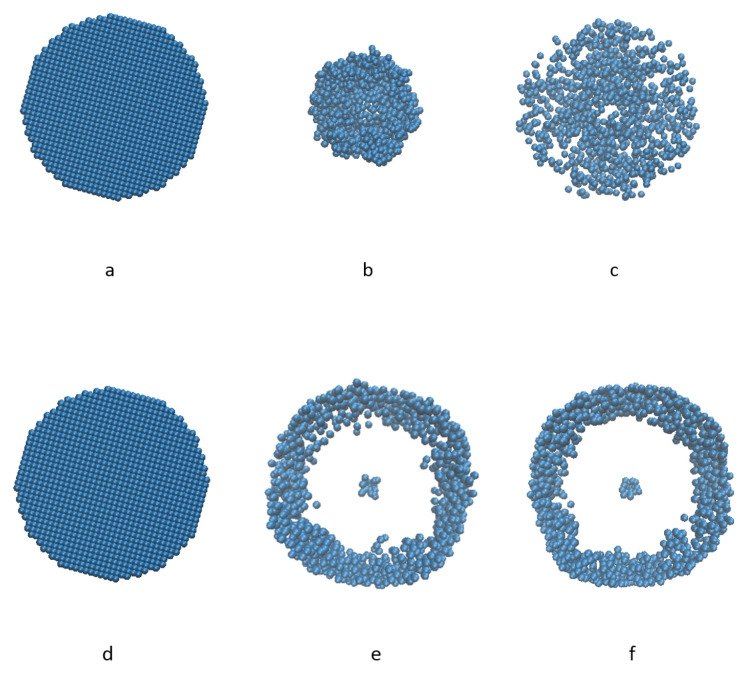
Liquid distribution comparison of high viscosity and low viscosity at different time (**a**) ν= 1 m${}^{2}$/s t = 0 (**b**) ν= 1 m${}^{2}$/s t = 2 s (**c**) ν= 1 m${}^{2}$/s t = 5 s (**d**) ν= 0.04 m${}^{2}$/s t = 0 (**e**) ν= 0.04 m${}^{2}$/s t = 2 s (**f**) ν= 0.04 m${}^{2}$/s t = 5 s.

A high viscosity inhabits the mixing process. When the viscosity of liquid is low, its effect on the mixing performance is not obvious, but when the viscosity of liquid is high, the viscous force between liquid and solid is also high. The liquid particles act like binders and bind solid particles together as local aggregates, which makes it hard to mix the solid particles completely. It is found that when the viscosity coefficient is small (0.004 and 0.2), the effect of viscosity on mixing index is not material. When the viscosity coefficient is 1 the mixing index curve differs significantly from the other two. The specific viscosity coefficient value for the mixing index curve to change dramatically should be explored in future. At the initial period, the mixing index is the same for all three cases, it is because at the initial stage the rotation speed of the blades is low and viscosity has not started to show its impact. In Figure 16, the three curves do not diverge until after a few seconds. The reason is that at the initial stage the rotation speed of the mixer is not very high and it also takes some time for the viscosity to shown its impact on the mixing index. However, even though a high viscosity decelerates the mixing progress, solid particles will mix well eventually given an enough mixing time.

In this part, a group of figures of liquid particles distribution are shown in Figure 16 from top view. When the viscosity of liquid particles is high, the viscous liquid will shrink towards the center first because of the rotation of the mixtures as in Figure 16b, and then extend to the rim gradually, eventually reach an almost homogeneous distribution horizontally, as in Figure 16c. On the other hand, when the viscosity of liquid is too low, all the liquid particles are distributed to the rim immediately and remain along the boundary for the rest of the simulation process. One can tell that there is no significant difference from Figure 16e–f. Appeared in the middle of the domain is a little liquid trapped by the blades, and the majority is distributed on the rim of the container. As shown in Figure 9 the distribution of solid particles is uniform in the radial direction, the case studies demonstrated that the liquid particles are hard to mixed homogeneously with the solid particles if the viscosity is too low.

### 4.5. The Effect Fluid Revolution on Mixing Behavior

In this section, we change the fluid resolutions to observe its effects on mixing Behavior. The smoothing length *h* has the physical meaning of constructing list for neighboring particles. Robinson and Rosswag pointed out that [64,65], there is an adaptive formula for selecting the smoothing length as Equation (30),

(30) $$\begin{array}{c} h=\eta \times {\left(\frac{m_{i}}{\rho_{i}}\right)}^{1/d} \end{array}$$

where η is a parameter with range of [1.2,1.5]; *d* is the dimensionality of the simulation problem; $m_{i}$ is the mass of the particle; and $\rho_{i}$ is local density. Combining Equations (15) and (30) and Table 4, smoothing length *h* could be simplified as Equation (31),

(31) $$\begin{array}{c} h=\eta {\left(\frac{1}{\sum_{liquid}W_{ij}+\sum_{solid}\frac{m_{j}}{m_{i}}W_{ij}}\right)}^{1/3} \end{array}$$

**Figure 17:**
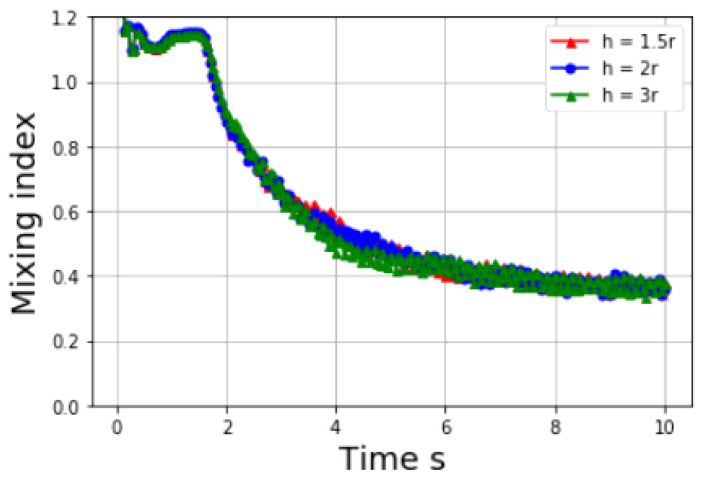
Mixing index at different smoothing length *h*.

Based on the situation that the solid load is high, therefore one can estimate by assigning the solid particles full influence, which yields *h* = [1.49r,1.86r], where *r* is the radius of the solid particle. Because the estimation is based on the solid particles occupy entire domain, and the *h* is constant during simulation, *h* beyond above range is expected. To investigate how smoothing length *h* influence the mixing index, a study is made with three different smoothing length, *h* = [1.5r,2r,3r]. Consistent with previous sections, materials with same properties in Table 4 are applied except the smoothing length. As Figure 17 shows, varying the smoothing length *h* will cause some fluctuations during the mixing process, however, it does not have strong effect on the final state. Therefore, one can conclude that under rational estimation with density, mass and DEM particle radius, the smoothing length *h* can be selected without affecting results.

## 5. Conclusions

The two-way SPH-DEM coupling method that covers the full spectrum of solid loads, especially for a high solid load, is presented to study high shear particle mixing problems. In this coupling scheme, a porosity is introduced for each SPH particle to calculate its density. The coupling force between SPH and DEM particles includes two parts: SPH pressure gradient equation is implemented to compute the pressure gradient force between DEM and SPH particles; whereas the viscous force is related to particles’ porosity by Darcy’s Law. In virtual experiments, two types of solid particles and one type of liquid particles are mixed in a four-bladed mixer. The mixing index measured by local average method is the main criterion to quantify the quality of the mixing status. The mixing processes with different proportions and viscosity values of the liquid are investigated. The virtual experiments with the present algorithm show the following aspects of the particle mixing process with high solid loads:

1. Adding more liquid can slower down the mixing process for a high solid load mix, and increasing the viscosity of the liquid will slow down the mixing process tremendously.
2. When visicosity of liquid reduces, although the solid particles can be mixed well eventually, the liquid distribution is not homogeneous, but concentrates at the edge due to the centrifugal effect.
3. A smoothing length at 1.5–2 times of the particle radius can provide convergent results of mixing index for virtual experiments.

Overall, the present SPH-DEM coupling method is stable and reasonable when the solid load is high, and this method could be applied to many kinds of solid mixed with a liquid. Particularly, for concrete production, fine aggregate, cement, and admixture powders are mixing with water. To obtain homogeneous mixture, as the physical experiments are expensive and time-consuming, replacing some physical tests with virtual experiments on computer may expedite the design process of concrete. Although the present method requires the calibration of parameters, it can reproduce the physical experiments on computer and advance the understanding of particle mixing. The model can be extended to other applications of particle systems.The present method can be extended to virtual experiments of particle mixing process with different mixer blades, solid particle densities and sizes, and liquid binder. One can use the method to formulate the mixing process and thus significantly expedite the material development cycles.

## Figures and Tables

**Table 1:** Parameters of the DEM and SPH particles in the sedimentation case study.

Parameter	Solid	Liquid
density	5000 kg/m${}^{3}$	1000 kg/m${}^{3}$
radius	0.5 m	h = 1 m
viscosity	30.8 m${}^{2}$/s	10 m${}^{2}$/s
numbers	1	30,740
velocity	0.218 m/s	0

**Table 2:** Parameters of particles in the case of a liquid flowing through a porous media case.

Parameter	Solid	Liquid
density	5000 kg/m${}^{3}$	1000 kg/m${}^{3}$
radius	0.5 m	h = 1 m
viscosity	depend on porosity	0.01 m${}^{2}$/s
volume fraction	52.36%	47.64%
numbers	1000	7479

**Table 3:** Geometric parameters of the high shear mixer.

Parameter	Value
Vessel diameter	400 mm
Vessel height	400 mm
Blade length	192 mm
Blade height	45 mm
Blade rake angle	$135^{\circ }$
Rotation speed	10 rad/s
Simulation time	20 s
Acceleration time	0.5 s
Time step	$5\times 10^{-5}$ s

**Table 4:** Parameters of solid and liquid particles in high shear mixing simulation.

Parameter	Solid
Diameter	10 mm
Density	1000 kg/m${}^{3}$
Mass	0.5236 g
Number	19,440
Normal Stiffness	$7\times 10^{7}$ N/m
Tangential Stiffness	$2\times 10^{7}$ N/m
Normal Damping Coefficient	8000 N· s /m
Tangential Damping Coefficient	4000 N· s /m
Coefficient of restitution	0.8
Friction coefficient	0.5
Parameter	Liquid
Diameter	10 mm
Density	1000 kg/m${}^{3}$
Mass	1 g
Number	1060
speed of sound	30 m/s
viscosity coefficient	0.2 m${}^{2}$/s

## Data Availability

The data presented in this paper can be obtained through the packages on our group website (Sustainable Engineering & Materials Laboratory). http://www.columbia.edu/cu/civileng/yin/software.html.

## Abbreviations

*W*, *h*	Kernel function, length width
*m*, ρ	mass and density
*g*	gravity acceleration
ν	Kinematic viscosity
*P*	Pressure
$F_{\mu }$, $F_{b}$	Viscous damping force and body force
$c_{0}$	Speed of the sound
I,ω, $T_{r}$	Momentum of inertia, rotation speed and torque of particle
$F_{n}$, $F_{t}$	Normal and tangential forces
$k_{n},k_{t}$	Spring stiffness along normal and tangential directions
$C_{n},C_{t}$	Damping coefficients along normal and tangential directions
$\Delta r_{n}$, $\Delta r_{t}$	Normal and tangential displacements
$m_{eff}$	Effective mass of particle
$e_{n}$	Collision coefficient
$P_{o}$, *V*	Porosity and volume
$F_{d}$	Viscous force
Φ	Volume fraction of the solid
$f_{ij}^{d,SPH}$	Drag force between particles *i* and *j*
$u_{k,l}$, $x_{k,l}$	Relative velocity and position between particles *k* and *l*
Δt	Time step
